# Supplementary material for: New records of non-indigenous species from the eastern Mediterranean Sea (Crustacea, Mollusca), with a revision of genus Isognomon (Mollusca: Bivalvia)
Source: PeerJ. 2024 May 31;12:e17425. doi: 10.7717/peerj.17425 (PMC11146324; doi:10.7717/peerj.17425)
Supplement: Supplemental Information 1 [file peerj-12-17425-s001.docx]

**New records of non-indigenous species from the eastern Mediterranean Sea (Crustacea, Mollusca), with a revision of genus *Isognomon* (Mollusca, Bivalvia)**

**Supplemental Information 1: additional tables**

Paolo G. Albano^1,2*^, Yuanyuan Hong^3^, Jan Steger^2^, Moriaki Yasuhara^3,4^, Stefano Bartolini^5^, Cesare Bogi^6^, Marija Bošnjak^7^, Marina Chiappi^8^, Valentina Fossati^8^, Mehmet Fatih Huseyinoglu^9^, Carlos Jiménez^8^, Hadas Lubinevsky^10^, Arseniy R. Morov^10^, Simona Noè^1,11,12^, Magdalene Papatheodoulou^8^, Vasilis Resaikos^8^, Martin Zuschin^2^, Tamar Guy-Haim^10^

^1^ Department of Marine Animal Conservation and Public Engagement, Stazione Zoologica Anton Dohrn, Naples, Italy

^2^ Department of Palaeontology, University of Vienna, Vienna, Austria

^3^ School of Biological Sciences, Area of Ecology and Biodiversity, Swire Institute of Marine Science, Institute for Climate and Carbon Neutrality, and Musketeers Foundation Institute of Data Science, The University of Hong Kong, Hong Kong SAR, China

^4^ State Key Laboratory of Marine Pollution, City University of Hong Kong, Kowloon, Hong Kong SAR, China

^5^ Firenze, Italy

^6^ Gruppo Malacologico Livornese, Livorno, Italy

^7^ Croatian Natural History Museum, Zagreb, Croatia

^8^ Enalia Physis Environmental Research Centre, Nicosia, Cyprus

^9^ Faculty of Maritime Studies, University of Kyrenia, Girne, Cyprus

^10^ Israel Oceanographic and Limnological Research, Haifa, Israel

^11^ Department of Integrative Marine Ecology, Stazione Zoologica Anton Dohrn, Naples, Italy

^12^ National Biodiversity Future Center, Palermo, Italy

Corresponding author:

Paolo G. Albano^1^

^1^ Villa Comunale, Naples, 80121, Italy

Email address: pgalbano@gmail.com

**Table S1: COI mtDNA sequences of *Striarca* specimens and outgroups included in the phylogenetic analysis.**

|  | **Species** | **Identifier** | **Accession number** | **Locality** | **Reference** |
| --- | --- | --- | --- | --- | --- |
| 1 | *Striarca* aff*. symmetrica* | BC015 | PP054322.1 | Israel, Mediterranean Sea | This study |
| 2 | *Striarca* aff*. symmetrica* | BC017 | PP054323.1 | Israel, Mediterranean Sea | This study |
| 3 | *Striarca* aff*. symmetrica* | BC018 | PP054324.1 | Israel, Mediterranean Sea | This study |
| 4 | *Striarca lactea* | SKIL2013_1 | PP029441.1 | Israel, Mediterranean Sea | This study |
| 5 | *Striarca lactea* | SKIL2013_2 | PP029442.1 | Israel, Mediterranean Sea | This study |
| 6 | *Striarca lactea* | CY535 | PP029441.1 | Cyprus, Mediterranean Sea | This study |
| 7 | *Striarca lactea* | CY062 | PP029442.1 | Cyprus, Mediterranean Sea | This study |
| 8 | *Striarca lactea* | BC081 | PP029437.1 | Cyprus, Mediterranean Sea | This study |
| 9 | *Striarca lactea* | CY052 | PP029438.1 | Cyprus, Mediterranean Sea | This study |
| 10 | *Striarca lactea* | CY138 | PP029439.1 | Cyprus, Mediterranean Sea | This study |
| 11 | *Striarca lactea* | CY165 | PP029440.1 | Cyprus, Mediterranean Sea | This study |
| 12 | *Striarca lactea* | BC033 | PP029432.1 | Crete, Greece, Mediterranean Sea | This study |
| 13 | *Striarca lactea* | BC035 | PP029433.1 | Crete, Greece, Mediterranean Sea | This study |
| 14 | *Striarca lactea* | BC036 | PP029434.1 | Crete, Greece, Mediterranean Sea | This study |
| 15 | *Striarca lactea* | BC037 | PP029435.1 | Crete, Greece, Mediterranean Sea | This study |
| 16 | *Striarca lactea* | BC043 | PP029436.1 | Puglia, Italy, Mediterranean Sea | This study |
| 17 | *Striarca lactea* | BC060 | PP029443.1 | Aroka, France, NE Atlantic | This study |
| 18 | *Striarca lactea* | BC061 | PP029444.1 | Aroka, France, NE Atlantic | This study |
| 19 | *Striarca lactea* | BC062 | PP029445.1 | Aroka, France, NE Atlantic | This study |
| 20 | *Striarca lactea* |  | KX713502.1 | Catalonia, Spain, Mediterranean Sea | Combosch et al. (2017) |
| 21 | *Striarca lactea* |  | MT920165.1 | Croatia, Mediterranean Sea | Buršić et al. (2021) |
| 22 | *Striarca symmetrica* | UF574617 (BOMAN-06654) |  | Oman, Persian Gulf | Florida Museum Collection |
| 23 | *Striarca symmetrica* | UF574910 (BOMAN-07357) |  | Oman, Persian Gulf | Florida Museum Collection |
| 24 | *Striarca symmetrica* | UF 584905 (BOMAN-13259) |  | Oman, Persian Gulf | Florida Museum Collection |
| 25 | *Striarca symmetrica* |  | MN608218.1 | Hainan, China | GenBank |
| 26 | *Striarca symmetrica* |  | MN608219.1 | Hainan, China | GenBank |
| 27 | *Striarca symmetrica* |  | MN608220.1 | Hainan, China | GenBank |
| 28 | *Arcopsis solida* |  | AF253493.1 | Panama, Pacific | Marko and Moran (2002) |
| 29 | *Arcopsis solida* |  | AF253477.1 | Panama, Pacific | Marko and Moran (2002) |
| 30 | *Arcopsis adamsi* |  | AF253482.1 | Panama, Caribbean Sea | Marko and Moran (2002) |
| 31 | *Arca noae* |  | KC429090.1 | Catalonia, Spain, Mediterranean Sea | Sharma et al. (2013) |

**Table S2: COI mtDNA and 16S rRNA sequences of *Isognomon* specimens and outgroups included in the phylogenetic analysis.**

|  | **Species** | **Identifier** | **Accession number** | **Locality** | **Reference** |
| --- | --- | --- | --- | --- | --- |
| **COI** |  |  |  |  |  |
| 1 | *Isognomon bicolor* | SKIL_1 | PP054325.1 | Israel, Mediterranean Sea | This study |
| 2 | *Isognomon bicolor* | PLIL_1 | PP054326.1 | Israel, Mediterranean Sea | This study |
| 3 | *Isognomon bicolor* | BC055 | PP054327.1 | Cyprus, Mediterranean Sea | This study |
| 4 | *Isognomon bicolor* | BC067 | PP054328.1 | Crete (Greece), Mediterranean Sea | This study |
| 5 | *Isognomon bicolor* |  | PP054329.1 | Florida, USA | This study |
| 6 | *Isognomon bicolor* |  | KX373613.1 | Florida, USA | Wilk (2016) |
| 7 | *Isognomon* aff. *legumen* | CY237 | PP054330.1 | Cyprus, Mediterranean Sea | This study |
| 8 | *Isognomon* aff. *legumen* | CY041 | PP054331.1 | Cyprus, Mediterranean Sea | This study |
| 9 | *Isognomon legumen* |  | AB076950.1 | Japan | GenBank |
| 10 | *Isognomon legumen* |  | MN608275.1 | China | GenBank |
| 11 | *Isognomon legumen* |  | MW284809.1 | Hawaii | GenBank |
| 12 | *Isognomon legumen* |  | KX713469.1 | Hong Kong | Combosch et al. (2017) |
| 13 | *Isognomon legumen* | UF574579 (BOMAN-06426) |  | Oman | Florida Museum Collection |
| 14 | *Isognomon legumen* | UF569885 (BOMAN-01236) |  | Oman | Florida Museum Collection |
| 15 | *Isognomon legumen* | UF569886 (BOMAN-01237) |  | Oman | Florida Museum Collection |
| 16 | *Isognomon legumen* | UF574574 (BOMAN-06420) |  | Oman | Florida Museum Collection |
| 17 | *Isognomon legumen* | UF570003 (BOMAN-01829) |  | Oman | Florida Museum Collection |
| 18 | *Isognomon legumen* | UF570164 (BOMAN-02926) |  | Oman | Florida Museum Collection |
| 19 | *Isognomon legumen* | UF574584 (BOMAN-06458) |  | Oman | Florida Museum Collection |
| 20 | *Isognomon legumen* | UF521351 |  | Saudi Arabia | Florida Museum Collection |
| 21 | *Isognomon legumen* | UF521049 (BDJRS-5539) |  | Saudi Arabia | Florida Museum Collection |
| 22 | *Isognomon legumen* | UF576272 (BOMAN-09347) |  | Oman | Florida Museum Collection |
| 23 | *Isognomon legumen* | UF577651 (BOMAN-12558) |  | Oman | Florida Museum Collection |
| 24 | *Isognomon legumen* | UF574566 (BOMAN-06394) |  | Oman | Florida Museum Collection |
| 25 | *Isognomon nucleus* | UF569863 (BOMAN-01130) |  | Oman | Florida Museum Collection |
| 26 | *Isognomon nucleus* | UF570233 (BOMAN-03596) |  | Oman | Florida Museum Collection |
| 27 | *Isognomon nucleus* | UF569864 (BOMAN-01131) |  | Oman | Florida Museum Collection |
| 28 | *Isognomon nucleus* | UF570234 (BOMAN-03597) |  | Oman | Florida Museum Collection |
| 29 | *Isognomon nucleus* | UF576676 (BOMAN-10467) |  | Oman | Florida Museum Collection |
| 30 | *Isognomon nucleus* | UF576677 (BOMAN-10468) |  | Oman | Florida Museum Collection |
| 31 | *Isognomon nucleus* | UF570232 (BOMAN-03595) |  | Oman | Florida Museum Collection |
| 32 | *Pinctada persica* |  | AB777259.1 | Iran, Persian Gulf |  |
| **16S rRNA** |  |  |  |  |  |
| 1 | *Isognomon bicolor* | SKIL_1 | PP034416.1 | Israel, Mediterranean Sea | This study |
| 2 | *Isognomon bicolor* | PLIL_1 | PP034417.1 | Israel, Mediterranean Sea | This study |
| 3 | *Isognomon bicolor* | PLIL_2 | PP034418.1 | Israel, Mediterranean Sea | This study |
| 4 | *Isognomon bicolor* |  | PP034419.1 | Florida, USA | This study |
| 5 | *Isognomon bicolor* |  | OK104096.1 | Italy | Garzia et al. (2022) |
| 6 | *Isognomon bicolor* |  | OK104097.1 | Italy | Garzia et al. (2022) |
| 7 | *Isognomon bicolor* |  | HQ329406.1 | Florida, USA | Tëmkin (2010) |
| 8 | *Isognomon* aff. *legumen* | CY041 | PP034420.1 | Cyprus, Mediterranean Sea | This study |
| 9 | *Isognomon* aff. *legumen* | CY237 | PP034421.1 | Cyprus, Mediterranean Sea | This study |
| 10 | *Isognomon recognitus* |  | KT317424.1 | Baja California Sur, Mexico | Raith et al. (2015) |
| 11 | *Isognomon recognitus* |  | KT317425.1 | Sonora, Guaymas, Mexico | Raith et al. (2015) |
| 12 | *Isognomon recognitus* |  | KT317426.1 | Sonora, Guaymas, Mexico | Raith et al. (2015) |
| 13 | *Isognomon recognitus* |  | KT317427.1 | Sonora, Puerto Penasco, Mexico | Raith et al. (2015) |
| 14 | *Isognomon alatus* |  | HQ329405.1 | Florida, USA | Tëmkin (2010) |
| 15 | *Isognomon alatus* |  | KC429251.1 | Florida, USA | Sharma et al. (2013) |
| 16 | *Isognomon alatus* |  | JN133622.1 | Puerto Rico | GenBank |
| 17 | *Isognomon ephippium* |  | KY081325.1 | Hainan, China | Liu et al. (2018) |
| 18 | *Isognomon* cf. *ephippium* |  | HQ329407.1 | Thailand | Tëmkin (2010) |
| 19 | *Isognomon radiatus* |  | HQ329408.1 | Florida, USA | Tëmkin (2010) |
| 20 | *Isognomon* sp. |  | HQ329409.1 | Clipperton Island | Tëmkin (2010) |
| 21 | *Pinctada maxima* |  | AB214435.1 | Philippines | Masaoka & Kobayashi (2005) |

# References

Buršić M, Iveša L, Jaklin A, Arko Pijevac M, Kučinić M, Štifanić M, Neal L, Bruvo Mađarić B. 2021. DNA barcoding of marine mollusks associated with *Corallina officinalis* turfs in southern Istria (Adriatic Sea). *Diversity* 13:196. DOI: 10.3390/d13050196.

Combosch DJ, Collins TM, Glover EA, Graf DL, Harper EM, Healy JM, Kawauchi GY, Lemer S, McIntyre E, Strong EE, Taylor JD, Zardus JD, Mikkelsen PM, Giribet G, Bieler R. 2017. A family-level Tree of Life for bivalves based on a Sanger-sequencing approach. *Molecular Phylogenetics and Evolution* 107:191–208. DOI: 10.1016/j.ympev.2016.11.003.

Garzia M, Furfaro G, Renda W, Rosati A-M, Mariottini P, Giacobbe S. 2022. Mediterranean spreading of the bicolor purse oyster, *Isognomon bicolor*, and the chicken trigger, *Malleus* sp., vs. the Lessepsian prejudice. *Mediterranean Marine Science* 23:777–788. DOI: 10.12681/mms.29218.

Liu J, Liu H, Zhang H. 2018. Phylogeny and evolutionary radiation of the marine mussels (Bivalvia: Mytilidae) based on mitochondrial and nuclear genes. *Molecular Phylogenetics and Evolution* 126:233–240. DOI: 10.1016/j.ympev.2018.04.019.

Marko PB, Moran AL. 2002. Correlated evolutionary divergence of egg size and a mitochondrial protein across the Isthmus of Panama. *Evolution* 56:1303–1309.

Masaoka T, Kobayashi T. 2005. Estimation of phylogenetic relationships in pearl oysters (Mollusks: Bivalvia: *Pinctada*) used for pearl production based on rRNA genes sequence. *DNA polymorphism* 13:151–162.

Raith M, Zacherl DC, Pilgrim EM, Eernisse DJ. 2015. Phylogeny and species diversity of Gulf of California oysters (Ostreidae) inferred from mitochondrial DNA. *American Malacological Bulletin* 33:263–283. DOI: 10.4003/006.033.0206.

Sharma PP, Zardus JD, Boyle EE, González VL, Jennings RM, McIntyre E, Wheeler WC, Etter RJ, Giribet G. 2013. Into the deep: A phylogenetic approach to the bivalve subclass Protobranchia. *Molecular Phylogenetics and Evolution* 69:188–204. DOI: 10.1016/j.ympev.2013.05.018.

Tëmkin I. 2010. Molecular phylogeny of pearl oysters and their relatives (Mollusca, Bivalvia, Pterioidea). *BMC Evolutionary Biology* 10:342. DOI: 10.1186/1471-2148-10-342.

Wilk JA. 2016. Evolution of the Isognomonidae Woodring, 1925: Phylogenetic and Morphometric Analyses. Chicago: Northwestern University.
